# Halloysite Nanotube-Enhanced Polyacrylonitrile Ultrafiltration Membranes: Fabrication, Characterization, and Performance Evaluation

**DOI:** 10.1021/acsomega.3c03655

**Published:** 2023-09-11

**Authors:** Seren Acarer, İnci Pir, Mertol Tüfekci, Tuǧba Erkoç, Sevgi Güneş Durak, Vehbi Öztekin, Güler Türkoǧlu Demirkol, Mehmet Şükrü Özçoban, Tuba Yelda Temelli Çoban, Selva Ćavuş, Neşe Tüfekci

**Affiliations:** † Faculty of Engineering, Department of Environmental Engineering, Istanbul University-Cerrahpasa, 34320 Istanbul, Avcilar, Turkey; ‡ Faculty of Mechanical Engineering, Istanbul Technical University, Istanbul 34437, Turkey; § Department of Mechanical Engineering, Imperial College London, South Kensington Campus, Exhibition Road, London SW7 2AZ, U.K.; ∥ Faculty of Engineering, Department of Chemical Engineering, Istanbul University-Cerrahpaşa, 34320 İstanbul, Avcilar, Turkey; ⊥ Department of Environmental Engineering, Faculty of Engineering-Architecture, Nevsehir Haci Bektas Veli University, Nevsehir 50300, Turkey; # Faculty of Civil Engineering, Yıldız Technical University - Davutpaşa, 34220 Istanbul, Turkey

## Abstract

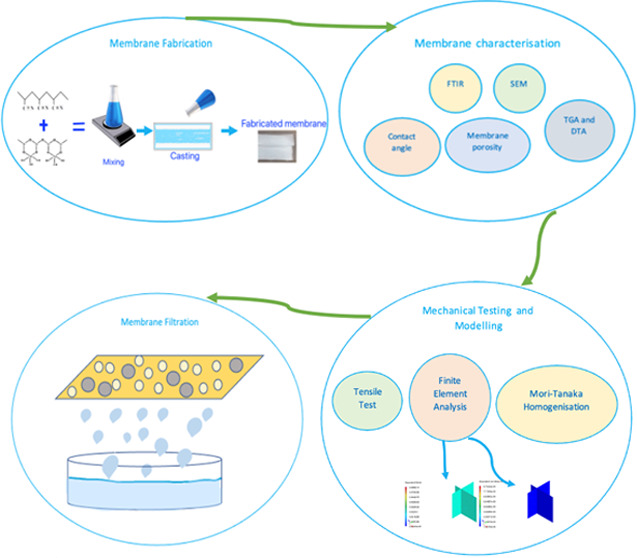

This research focuses on the production and characterization of pristine polyacrylonitrile (PAN) as well as halloysite nanotube (HNT)-doped PAN ultrafiltration (UF) membranes via the phase inversion technique. Membranes containing 0.1, 0.5, and 1% wt HNT in 16% wt PAN are fabricated, and their chemical compositions are examined using Fourier transform infrared (FTIR) spectroscopy. Scanning electron microscopy (SEM) is utilized to characterize the membranes’ surface and cross-sectional morphologies. Atomic force microscopy (AFM) is employed to assess the roughness of the PAN/HNT membrane. Thermal characterization is conducted using thermal gravimetric analysis (TGA) and differential thermal analysis (DTA), while contact angle and water content measurements reveal the hydrophilic/hydrophobic properties. The pure water flux (PWF) performance of the porous UF water filtration membranes is evaluated at 3 bar, with porosity and mean pore size calculations. The iron (Fe), manganese (Mn), and total organic carbon (TOC) removal efficiencies of PAN/HNT membranes from dam water are examined, and the surfaces of fouled membranes are investigated by using SEM post-treatment. Mechanical characterization encompasses tensile testing, the Mori–Tanaka homogenization approach, and finite element analysis. The findings offer valuable insights into the impact of HNT doping on PAN membrane characteristics and performance, which will inform future membrane development initiatives.

## 1 Introduction

The pursuit of efficient water and wastewater treatment techniques has become a global priority due to increasing concerns about water scarcity and environmental pollution. Membrane-based separation technologies have emerged as promising solutions offering both efficiency and versatility. Among these, pressure-driven membrane processes have gained significant attention for their widespread application in water and wastewater treatment. These membranes, which can be either organic (polymeric) or inorganic, are distinguished by the materials used in their fabrication. Polymeric membranes, particularly those made from polyacrylonitrile (PAN), are favored over inorganic counterparts due to their ease of production, superior mechanical properties, and cost-effectiveness.

Pressure-driven membrane processes are the most widely used membrane processes in water and wastewater treatment.^1,2^ Such membranes are classified as organic (polymeric) or inorganic according to the material used in their production.^3^ Polymeric membranes are more widely used in water treatment compared to inorganic membranes due to their ease of manufacture, mechanical properties, and low cost.^4^ PAN as a polymer matrix material has important characteristics such as low cost;^5,6^ good mechanical^6,7^ and impact strength;^7^ and remarkable chemical,^6,8^ thermal,^6−8^ and heat resistance.^7^ PAN membranes prepared using nanosized inorganic materials exhibit enhanced thermal and mechanical properties.^9^ Zhai et al. reported that the use of PAN enhanced the thermal stability and mechanical features of composite films.^7^ Naseeb et al. prepared PAN-graphene oxide-silicon dioxide membranes and reported that PAN and nanofiller together increased the chemical and mechanical stability and performance of the hybrid membrane.^10^

Due to its superior properties, PAN is widely used in the preparation of pressure-driven microfiltration (MF), ultrafiltration (UF), nanofiltration (NF), and reverse osmosis (RO) membranes.^11^ In addition, the relatively more hydrophilic nature of PAN contributes to its lower fouling tendency compared to polymers such as poly(ether sulfone) (PES), polysulfone (PSF), and polyethylene (PE) used in membrane preparation. Therefore, PAN is a good alternative membrane material for these other polymers used in membrane production.^12,13^ DMSO, a green solvent, is nontoxic and easier to recycle than traditional solvents.^14^

Rana et al. examined the miscibility behavior of poly(phenyl acrylate) (PPA) and poly(vinyl benzoate) (PVBZ) with poly(styrene-*co*-acrylonitrile) (SAN) using analog calorimetry, highlighting the differential heat of mixing of their low-molecular-weight analogs.^15^ In another study by Bhattacharya et al., the thermodynamic characteristics of miscible blends from polymers such as poly(ethyl acrylate) and poly(vinyl propionate) were explored through inverse gas chromatography.^16^ Rana et al. also investigated the miscibility and phase diagrams of PPA and SAN blends, accounting for solvent effects and binary polymer segment interaction parameters.^17^ Last, Rana et al. reported on the miscibility of poly(ethyl methacrylate) (PEMA) and poly(styrene-*co*-butyl acrylate) (SBA) and observed the existence of both upper critical solution temperature (UCST) and lower critical solution temperature (LCST).^18^

Membranes prepared using conventional materials often suffer from low flux, low selectivity, and high fouling tendency.^19,20^ Mixed matrix membranes (MMMs) are prepared by incorporating inorganic nanomaterials into the polymer matrix to take advantage of the processability, economic advantages, and superior properties of inorganic materials.^19,21−23^ HNTs (Al_2_Si_2_O_5_(OH)_4_*n*H_2_O) are naturally occurring, nontoxic, biocompatible clay minerals with a chemical composition like kaolinite, composed of aluminum, silicon, hydrogen, and oxygen.^24,25^ The diameters of HNTs are less than 100 nm, and their length can be up to several μm.^25,26^ The high surface area (∼65 m^2^/g),^27^ high aspect ratio (*L*/*D* = 10–50), high Young’s modulus (∼140 GPa), and low cost (∼4 $/kg)^28^ of HNTs make them ideal nanomaterials for the fabrication of polymer-based nanocomposites.

Many researchers reported that the incorporation of HNT into the polymer-based membrane matrix improves membrane surface wettability/hydrophilicity,^29−36^ porosity,^29,30,32,35,37,38^ membrane flux performance,^29−33,35−37^ mechanical properties, and^29,30,34^ resistance to fouling.^30,39^ In the study of Park et al., the PWF and tensile strength of the PES membrane changed from 7.1 to 24 L/m^2^ h and 4.4 to 5.2 MPa, respectively, with 0.5% HNT contribution to the membrane.^29^ In the study by Kamal et al. found that the modulus of elasticity of the pure PSF membrane, which is 142 MPa, increased by 15.6% to 164.1 MPa with the addition of 0.2% HNT to the membrane.^30^

Rana et al. investigated the thermal and mechanical properties of binary blends of metallocene PE with conventional polyolefins, revealing that all blend systems, while thermodynamically immiscible, are mechanically compatible with varying degrees of compatibility depending on their chemical structures.^40^ Later, Rana et al. studied the rheological and morphological behaviors of commercially available binary blends of ethylene 1-octene copolymer (EOC), discovering that miscibility and phase behavior were influenced by the melt index, density, and comonomer content.^41^ In subsequent work, Rana et al. examined the thermal, viscoelastic, and mechanical behaviors of EOC binary blends, noting a phase separation in crystallization but miscibility in the amorphous region.^42^ In a different context, Rapp et al. studied cross-linked linear PE/branched PE blends under thermo-oxidation, finding that accelerated ageing tests using the Arrhenius approach may lead to critical errors when predicting polymer lifetime.^43^

The determination of the mechanical properties of membranes has a significant role in membrane system design due to water pressure. The mechanical properties of membranes can be determined theoretically and experimentally. There are studies on the determination of mechanical properties of composite materials with experimental and numerical techniques in the literature.^44−49^ The tensile test is an example of the experimental processes, and the homogenization method is an example of the numerical study. With the homogenization method, membrane behavior can be predicted without applying any mechanical test as long as the mechanical properties of the individual matrix and reinforcing phases are known. This enables the prediction of whether the mechanical properties of the newly formed composite membrane structure will be at the desired values. It also contributes to time-saving and economic savings in membrane technologies. Furthermore, hygrothermal effects on material behavior also have a significant role in membrane service life regarding membrane working conditions. To obtain the mechanical properties of the material, experiments can be performed under different hygrothermal conditions.^50^ Besides, strain rate may also have a significant impact on the mechanical properties of the polymer-based composites.^51^ This approach can be employed in membrane testing processes. There are similar studies on the mechanical properties of membranes under wet and dry conditions.^52−55^ Based on previous research, it is possible to state that the stiffer tubular structures like HNT contribute to the mechanical stiffness and strength significantly.^51,56,57^

In this study, the effects of HNT addition on the physical, chemical, thermal, and mechanical properties as well as the water flux performance of the PAN membranes produced by phase inversion are investigated. Different HNT amounts (0.1, 0.5, and 1 wt %) are incorporated into the PAN membranes to determine the most suitable HNT concentration that provides the best properties and performance. Membranes are fabricated using the phase inversion method and characterized through Fourier transform infrared (FTIR) spectroscopy, scanning electron microscopy (SEM), contact angle measurements, and water content determination. Membrane porosity and mean pore size are calculated, while thermal properties are analyzed through differential thermal analysis (DTA) and thermal gravimetric analysis (TGA). Furthermore, PWF performance and membrane treatment performance in iron (Fe), manganese (Mn), and total organic carbon (TOC) removal from dam water are investigated. After the dam water filtration, the surfaces of the fouled membranes are examined by SEM analysis and the membrane that was more resistant to fouling is determined. Finally, the mechanical properties are explored via tensile testing, finite element analysis, and the Mori–Tanaka homogenization method. To the authors’ knowledge, there are no studies on the characterization of flat-sheet type PAN membranes with varying amounts of HNT added.

This study presents a novel approach to the development of flat-sheet-type PAN membranes incorporating HNT, which has not been explored in previous research. The comprehensive investigation of the effects of HNT addition on PAN membranes’ physical, chemical, thermal, and mechanical properties, along with their water flux performance, contributes significantly to the understanding of HNT-PAN membrane systems. The findings from this study offer valuable insights for the optimization of PAN membrane production, potentially paving the way for innovative applications in water treatment and other related fields.

## 2 Materials and Methods

### 2.1 Materials

PAN (average Mw = 150 000 g/mol) and DMSO were purchased from Sigma-Aldrich. HNT (diameter: 30–70 nm, length: 1–3 μm) was purchased from Nanografi Co. Ltd. All solvents and materials are used as purchased without further purification.

### 2.2 Membrane Fabrication by Phase Inversion

Neat PAN and HNT-doped PAN membranes are fabricated by the phase inversion method. The neat PAN membranes are fabricated at 16% wt. Based on previous experiences^58,59^ with membrane fabrication and characterization, the low polymer content of the membrane casting solution results in insufficient mechanical strength of the fabricated membrane due to the low viscosity of the casting solution. Since the mechanical strength of FS-doped membranes containing 16% wt of PAN in a previous study^59,60^ is quite suitable for the characterization of the membranes, the PAN ratio in the membranes is kept as 16% wt in this study. PAN is added to DMSO and mixed using a magnetic stirrer with a heater (WiseStir MSH*-*20A) for 24 h at 60 °C until a homogeneous solution is obtained. Before starting the membrane casting, the solution bottles are kept in an ultrasonic bath (Weightlab Instruments) at 25 °C for 30 min to remove the bubbles in the casting solution. Membrane solution is poured onto the glass layer, and polymeric films are formed using a 200 μm-thick blade (TQC Sheen, VF2170-261). Subsequently, the glass layer is immersed in a coagulation bath with distilled water. Membrane fabrication is completed as a result of the exchange of solvent (DMSO) in the casting solution and nonsolvent (distilled water) in the coagulation bath. The fabricated membranes are kept in distilled water at 4 °C for 24 h before characterization.

For the fabrication of HNT-doped nanocomposite PAN membranes, the required amount of HNT (0.1–1% wt) is added into DMSO and dispersed for 30 min at 60 °C in a magnetic stirrer. Then, PAN is added and mixed for 24 h until a homogeneous solution is obtained. In the next steps, the same procedures are applied to the neat PAN membrane for the fabrication of HNT-doped membranes. In the next steps, the same processes applied to the neat PAN membrane are applied to the production of the PAN-HNT nanocomposite membranes. Table 1 shows the composition of the membrane casting solutions and the codes of the fabricated membranes.

**Table 1:** Composition of the Membrane Casting Solutions

**membrane code**	**PAN** (wt %)	**DMSO** (wt %)	**HNT** (wt %)
Neat PAN	16	84.0	
PAN-HNT-0.1	16	83.9	0.1
PAN-HNT-0.5	16	83.5	0.5
PAN-HNT-1	16	83.0	1

### 2.3 Membrane Characterization

#### 2.3.1 FTIR

The functional groups of neat PAN and PAN-HNT membranes are determined using FTIR spectroscopy. The FTIR spectra of membrane samples are obtained using FTIR spectrometry (PerkinElmer Spectrum 100) in the wavenumber range of 4000–650 cm^–1^.

#### 2.3.2 SEM

Surface and cross-sectional images of the membranes are obtained using SEM (FEI Quanta 250 FEG). To take SEM images of insulating polymeric membranes, first, the membranes are made conductive by coating them with gold. SEM images of the membranes are observed at 20 kV. While the SEM surface views of the membranes are examined at 5000× magnification, the cross-sectional views are examined at 1500× magnification. SEM surface images of fouled PAN/HNT membranes after the dam water was filtered are examined under 10,000× and 5000× magnification.

#### 2.3.3 AFM

The morphological characterization of the prepared membrane is carried out using atomic force microscopy (AFM, Digital Instruments). AFM analysis of the dry PAN-HNT-0.5 membrane sample is performed in contact mode and with a scanning area of 10 μm × 10 μm. A silicon nitride probe (Bruker) is used in AFM analysis. After the analysis, in addition to examining the membrane surface morphology, numerical values corresponding to the roughness parameters are also determined.

#### 2.3.4 Water Content

To determine the water content of the membranes, all membrane samples are prepared by cutting them to the same size. The membranes are kept in an oven at 60 °C (NUVE EN 500) for 24 h, and then their dry weight is determined using a precision balance (KERN 573). Subsequently, the membranes are submerged in distilled water, and the weight of the damp membranes is ascertained immediately after the excess water on them is eliminated using drying paper. The water content of the membranes is computed utilizing eq 1.
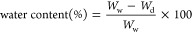
1where *W*_w_ and *W*_d_ are the wet and dry weights of membranes (g), respectively.

#### 2.3.5 Porosity

The porosity (ε) of the membranes is determined by the gravimetric method and calculated by eq 2
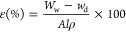
2where *W*_w_ is the wet weight of the membrane (g), *W*_d_ is the dry weight of the membrane (g), *A* is the membrane area (cm^2^), *l* is the membrane thickness (cm), and ρ is the density of the water (0.998 g/cm^3^).

#### 2.3.6 Mean Pore Size

The mean pore size (*r*_m_) of the membranes is calculated using the Guerout–Elford–Ferry equation given in eq 3
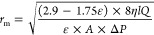
3where ε is the membrane porosity, η is the viscosity of water (8.9 × 10^–4^ Pa.s), *l* is the membrane thickness (m), *Q* is the volume of permeate water per unit time (m^3^/s), *A* is the effective membrane area (m^2^), and Δ*P* is the operating pressure (0.3 MPa).

#### 2.3.7 Contact Angle

The surface hydrophilicity of the membranes is determined using a contact angle goniometer (KSV CAM-101). Before measuring the contact angle of the membranes, the membranes are dried at room temperature for 2 h. Distilled water is dropped with a syringe to different positions of the membrane surface, and the angle between the water drop and the membrane surface is determined. Contact angle measurements are made three times for each membrane sample, and the results are given as averages.

#### 2.3.8 Thermal Study

The thermal analysis of the prepared membranes is performed using the DTA-TGA apparatus (Shimadzu DTG-60) with a heating rate of 10 °C min^–1^ between a temperature range of 25 and 700 °C in a nitrogen gas atmosphere.

#### 2.3.9 Pure Water Flux

A dead-end filtration system (Tin Mühendislik) is used to determine the PWF of the membranes. After the 5 cm-diameter membranes are placed in the filtration cell, they are compressed using N_2_ gas at 5 bar pressure for 10 min. Then, pure water is passed through the membranes at a pressure of 3 bar. The permeate is collected in a beaker on a precision balance (AND EJ-610). Data on time and permeate weight are transferred to the computer. The PWF of the membranes is calculated using eq 4.
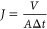
4where *J* is the membrane flux (L/m^2^ h), *V* is the permeate volume (L), *A* is the effective membrane area (m^2^), and Δ*t* is the time (h).

### 2.4 Membrane Treatment Performance

In this study, the dam water filtered from the membranes is collected from the Akçay Dam located in Sakarya, Turkey. Dam water is filtered from PAN/HNT membranes using a dead-end filtration cell (Tin Mühendislik) to test the performance of the membranes in Fe, Mn, and TOC removal. The same pressure used in PWF filtration is used for dam water filtration through membranes. PerkinElmer ICP-OES device is used to determine Fe and Mn removal, and a TOC analyzer (Shimadzu TOC-VCPN) is employed to determine TOC removal from the dam water by membranes. TOC measurements are performed according to Combustion Infrared Method 5310 B specified in the Standard Methods (APHA, 1998). The characterization of the dam water sample used in the filtration studies is shown in Table 2.

**Table 2:** Characterization of Dam Water

**parameter**	**unit**	**value**
Fe	mg/L	0.46 ± 0.04
Mn	mg/L	0.04 ± 0.007
pH		7.39 ± 0.05
Turbidity	NTU	1.53 ± 0.04
Total hardness	mg/L CaCO_3_ (°F)	9.70 ± 0.10
TOC	mg/L	1.85 ± 0.12

### 2.5 Mechanical Testing and Modeling of Membranes

Mechanical modeling of membranes can be done with several methods and plays a significant role in the designs of membrane systems regarding the membrane service life, operating conditions, etc. In this study, tensile testing is used to characterize the membrane’s mechanical behavior. To model the mechanics of the membranes, the Mori–Tanaka homogenization method and finite element analysis are employed.

#### 2.5.1 Tensile Test

On tensile testing, an axial load is applied to the membrane specimen, and force and stroke data are measured until the failure of the specimen. From the measured data, the mechanical behavior of the membrane specimen can be obtained. In this study, the strain rate for quasistatic testing is established to be less than 1% strain per minute. All membrane combinations are examined under both wet and dry conditions, and tests are carried out three times to guarantee repeatability. Tensile tests are applied with the Shimadzu AG-IS 50 kN universal test machine.

#### 2.5.2 Numerical Modeling

In order to keep costs such as production, time, and experiment costs to a minimum and obtain information about the porous material structure, numerical analysis modeling is needed as a preliminary study. This study provides a more effortless and quick comparison of the material to be designed and provides predictability. With this predictability, expensive and time-consuming production and testing processes can be used more effectively. There are various techniques accepted in the literature. In this study, to predict the mechanical behavior of the HNT-doped PAN membrane Mori–Tanaka homogenisation method, finite element analysis using fast Fourier transform (FFT) is performed.

Within the Mori–Tanaka homogenization approach, the mechanical properties of the composite material are computed utilizing a closed-form analytical equation based on the mechanical properties of the constituent materials. Using an analytical methodology, the Mori–Tanaka method is computationally cheap, accurate, and easy to use.^48^ The mechanical properties of the PAN membrane are introduced to the software as a representative volume element (RVE), and thus, a model is established. The analyses are then completed by applying uniaxial stress to this RVE.

In order to ascertain the mechanical properties of composite membranes, finite element analysis is utilized as well. Like the Mori–Tanaka homogenization method, an RVE is selected.^61^ Periodic boundary conditions on this RVE are assumed, and the finite element model is solved by using the FFT-based approach. Due to the periodicity assumption and its efficiency in representing the material with its structure while generating the RVE, the size and content of the RVE should be chosen conscientiously.^62−65^ This approach is computationally more reasonable compared to conventional finite element models; however, it is still more costly than Mori–Tanaka.

## 3 Results and Discussion

### 3.1 Characterization of PAN and PAN/HNT Membranes

#### 3.1.1 FTIR Analysis

**Figure 1:**
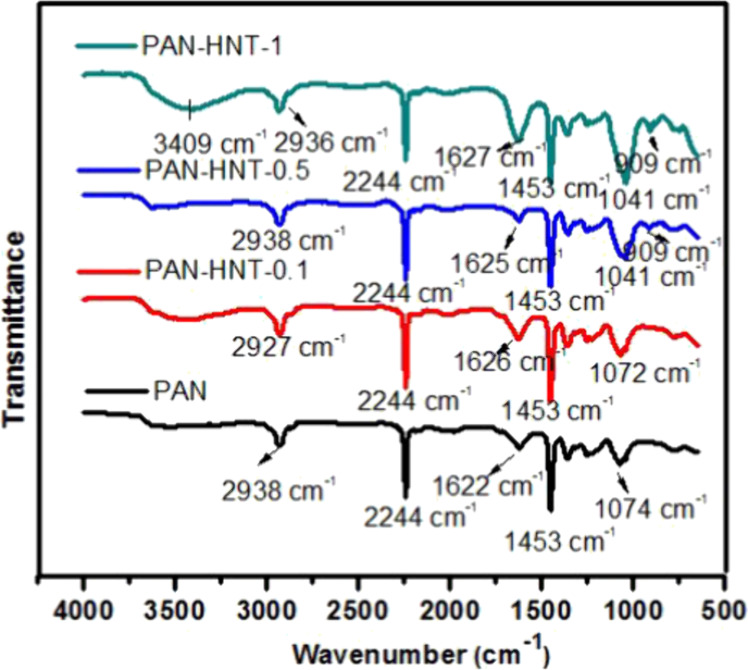
FTIR spectra of neat PAN and HNT-doped PAN membranes.

The FTIR spectra of membranes (PAN, PAN-HNT-0.1, PAN-HNT-0.5, and PAN-HNT-1) are presented in Figure 1. The characteristic peaks at 2244 and 1453 cm^–1^ are related to the strong −C≡N stretching^6,66^ and bending of C–H in CH_2_ for PAN,^6^ respectively. C–H stretching^6^ of membranes can be seen at 2938, 2927, 2938, and 2936 cm^–1^ for PAN, PAN-HNT-0.1, PAN-HNT-0.5 and PAN-HNT-1, respectively. It is reported that HNT showed main peaks at 3695 cm^–1^,^9,67^ 3621 cm^–1^,^9^ 1631 cm^–1^,^68^ 1118 cm^–1^,^67^ 999 cm^–1^,^69^ 905 cm^–1^,^9^ 791 cm^–1^,^67^ and 554 cm^–1^^68^ due to the O–H stretching vibration of inner-surface hydroxyl groups, O–H stretching vibration of inner hydroxyl group, deformation vibration of interlayer water, perpendicular Si–O stretching, Si–O–Si stretching vibration, O–H deformation of inner hydroxyl groups, symmetric stretching of Si–O, and deformation vibration of Al–O–Si, respectively. It is observed that the peak associated with the O–H deformation of inner hydroxyl groups of HNT exists at 909 cm^–1^ in the PAN/HNT membranes, except for PAN-HNT-0.1. The shift in the band from 1074 to 1041 cm^–1^ can be ascribed to hydrogen bond interactions between the O–H groups of HNT and the C–N groups of PAN for PAN-HNT-0.5 and PAN-HNT-1. These results are consistent with the literature.^9^ The FTIR results of PAN-HNT-0.1 and neat PAN are similar, probably due to the lower HNT content of PAN-HNT-0.1. It is clear that the peak intensities at 1627 and 1041 cm^–1^ increased in the spectrum of PAN-HNT-1. In addition, a broadening of the peak is observed at 3409 cm^–1^ for PAN-HNT-1. It is reported that when the HNT content increases, the peak intensity of the −OH increases owing to the Al–OH groups on the inner surface of HNT.^70^ The peak intensities at 1625 and 3409 cm^–1^ decrease more in the spectrum of PAN-HNT-0.5 compared to those of other membranes. This can be attributed to the good dispersion of HNT in the PAN-HNT-0.5 membrane, as observed in the TGA and SEM characterizations.

#### 3.1.2 Morphological Characterization

##### 3.1.2.1 SEM Images of HNT

**Figure 2:**
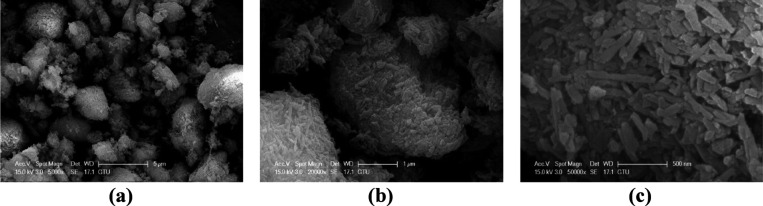
Scanning electron images of pure HNT magnified at (a) 5.00K, (b) 20.00K, and (c) 50.00K X.

SEM images of a pure halloysite nanotube at different magnifications are shown in Figure 2. Halloysite nanotubes have increasing attention with their hollow tubular structure. Despite the similarity of its structure to a carbon nanotube, halloysite nanotubes have distinctive advantages such as low price and perfect biocompatibility.^71^ It is previously reported that halloysite nanotubes have rodlike and tubular structures,^72^ and the length and inner and outer diameters of the halloysite nanotubes are described.^71^ SEM confirms that halloysite nanotubes are rodlike-shaped, and the dimensions are also compatible with the literature.

##### 3.1.2.2 SEM Images of PAN/HNT Membranes

**Figure 3:**
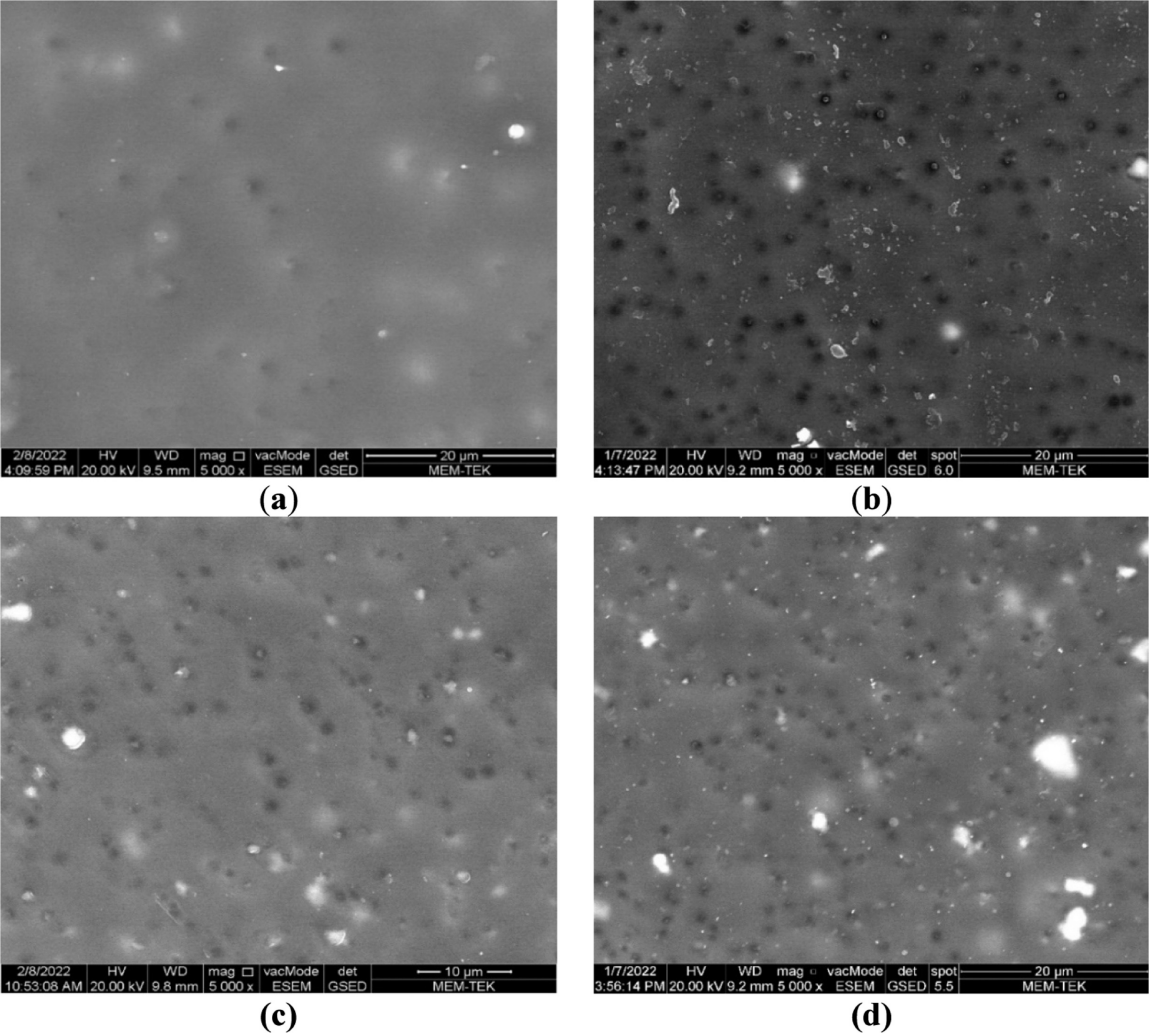
SEM surface views of (a) neat PAN, (b) PAN-HNT-0.1, (c) PAN-HNT-0.5, and (d) PAN-HNT-1.

Figure 3 shows SEM surface views of neat PAN and HNT-doped nanocomposite PAN membranes. All membranes exhibited a porous structure. The surface porosity of HNT-doped membranes is higher than that of neat PAN membranes. Even a very low amount of HNT (0.1 wt %) added to the PAN membrane resulted in a significant increase in membrane surface porosity. This phenomenon may be associated with the increase in membrane porosity, as hydrophilic −OH groups in HNT accelerate the exchange rate between solvent (DMSO) and nonsolvent (water) during phase inversion.^29,32^

**Figure 4:**
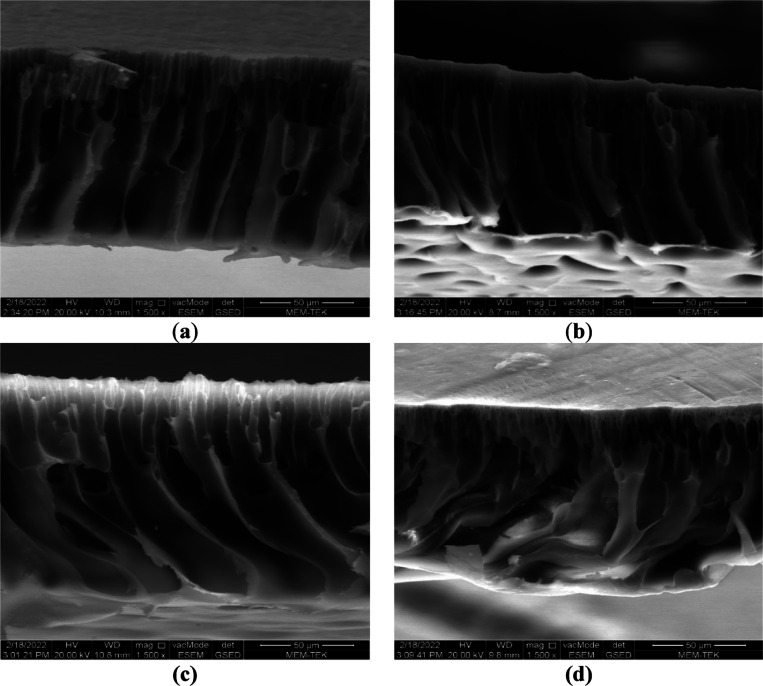
SEM cross-sectional views of (a) neat PAN, (b) PAN-HNT-0.1, (c) PAN-HNT-0.5, and (d) PAN-HNT-1.

Figure 4 shows SEM cross-sectional views of the PAN and HNT-doped PAN membranes. Neat PAN and PAN-HNT membranes exhibited an asymmetrical structure with a dense top layer responsible for selectivity and a bottom layer consisting of macro-voids and finger-like pores. HNT addition is caused by a change in finger-like pore size and pore amount of the PAN membrane. When the PAN and PAN-HNT-0.1 membranes are compared, it is clearly seen that horizontally extending macropores are formed in the lower layer of the membrane with the addition of HNT. This shows that the addition of a low amount of HNT into the PAN membrane is effective in changing the internal structure of the membrane. When HNT continued to be added to the PAN membrane above 0.1% wt, the horizontally extending macropores in the bottom layer began to disappear, and the horizontally extending macropores in the PAN-HNT-1 membrane disappeared completely. The increased viscosity of the membrane casting solution with the addition of nanomaterials can change the morphology of the membrane by changing the liquid–liquid exchange rate during phase inversion.^32,38^ As a result of the high amount of HNT added to the membrane casting solution (1% wt), the exchange rate slowed down during the phase inversion due to the excessive increase in the viscosity of the casting solution, and the internal structure of the membrane became denser.^73^ In addition, when SEM images of neat PAN and HNT-doped PAN membranes are compared, it can be concluded that HNT is better dispersed in the PAN-HNT-0.5 membrane.

#### 3.1.3 AFM

**Figure 5:**
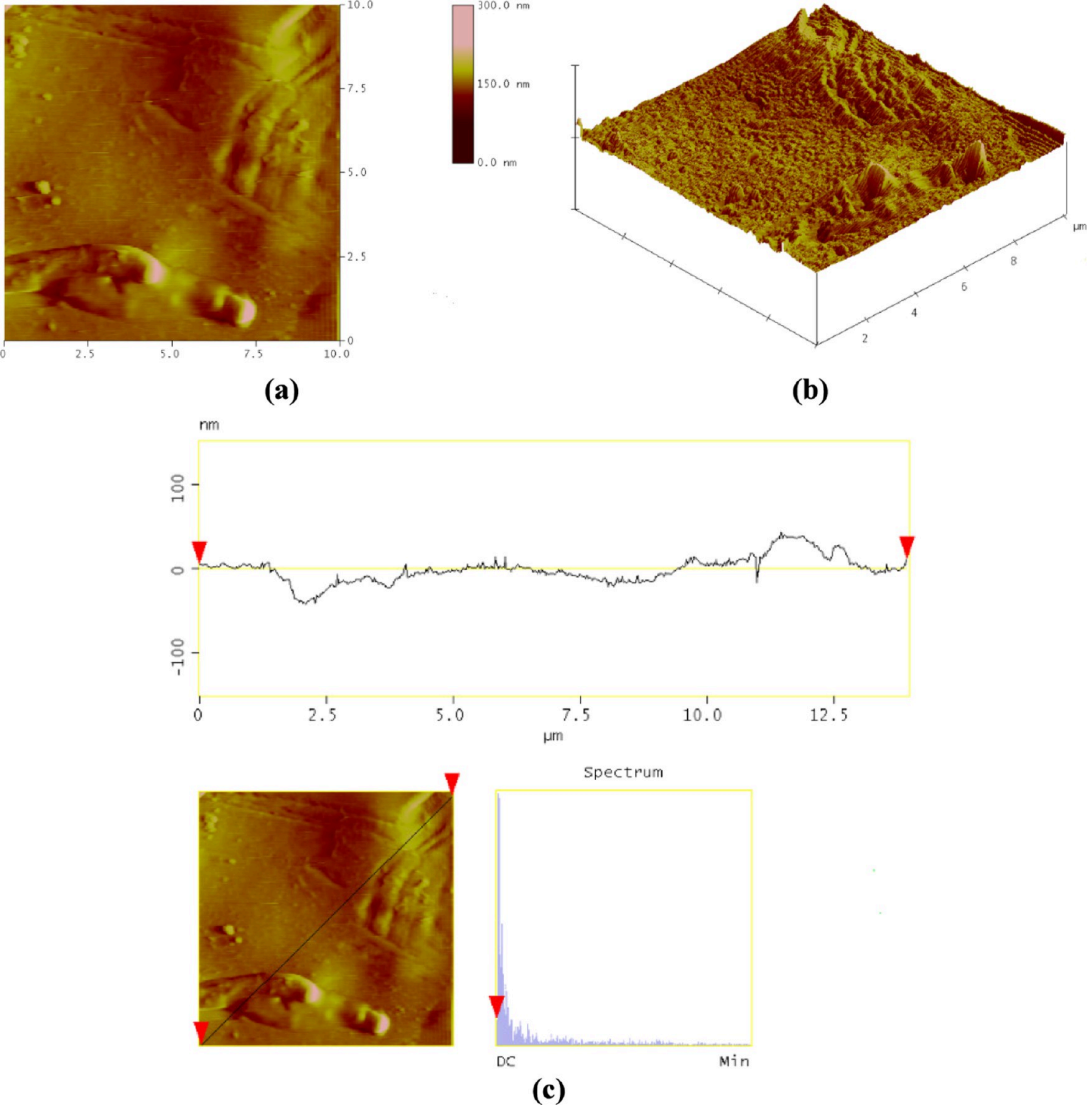
AFM images of the PAN-HNT-0.5 (a) two-dimensional (2D) mode, (b) three-dimensional (3D) mode, and (c) height profile.

AFM analysis is used to determine the topographic structure and surface roughness of PAN-HNT-0.5. Figure 5 shows the 3D and surface AFM images of the PAN-HNT-0.5 membrane. The lighter and darker colors on the membrane surface correspond to a higher position (for the membrane surface) and membrane pores, respectively.^74^

The higher mean roughness value can be attributed to the presence of higher HNT content on the membrane surface.^36^ The mean roughness (*R*_a_) value for PAN-HNT-0.5 was 9.40 nm. The root-mean-square roughness (*R*_q_) of PAN-HNT-0.5 is 13.15 nm (Figure 5). The maximum height of the roughness profile (*R*_*z*_) of the PAN-HNT-0.5 membrane is 34.81 nm (Figure 5). Adding a larger content of nanomaterial results in increased roughness and the possibility of agglomeration by halloysite. It is also reported that the use of lower contents of HNTs provides better dispersion of nanomaterial on the membrane’s surface.^36^

The pore structures of the membrane can be affected by the presence of nanostructures, such as nanocellulose. In the study of Kian et al., the use of nanocellulose particles provided better pore formation in the polymer matrix.^75^ It is reported that the average roughness of the membrane increased with the addition of the nanoparticle to the membrane owing to its homogeneous distribution on the membrane surface. It is stated that when silica was used in membranes (compared to bentonite), the surface roughness decreased due to the increase in elastic modulus.^76^ In the current study, the PAN-HNT-0.5 membrane showed a well-designed porous structure, homogeneous distribution, and suitable surface roughness.

#### 3.1.4 Membrane Porosity and Mean Pore Size

**Figure 6:**
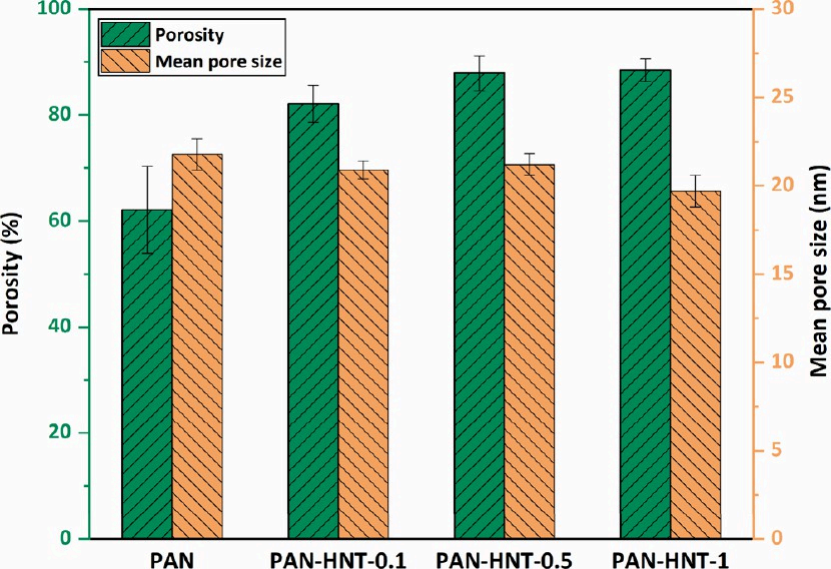
Porosity and mean pore size of neat PAN and HNT-doped PAN membranes.

The weight difference method is a method used to compare the porosity of the membrane under the same conditions.^77^Figure 6 shows the porosity and mean pore size results of the membranes. The porosity of HNT-doped membranes is higher than that of the neat PAN membrane (62.1% ± 8.2%). Porosity gradually increased up to 0.5% wt HNT addition to the membranes, but the increase in porosity lost its significance at 1% wt HNT addition. This situation is in harmony with the increase in the size of finger-like pores in both the surface and the inner structure until 0.5% HNT addition, and with the addition of 1% HNT, the inner structure becomes dense and the number and size of finger-like pores decrease (Figure 4c,d).

The mean pore sizes of the membranes range from 19.6 ± 0.9 to 21.7 ± 0.9 nm, which is included in the pore size range of UF membranes.^78^ Mean pore size decreased with the incorporation of HNT into the membranes. The lower mean pore size of the PAN-HNT-1 membrane compared to other membranes can be explained by the fact that high amounts of HNT cause blockage in the pores of the membrane.^38^

#### 3.1.5 Membrane Surface Hydrophilicity and Water Content

**Figure 7:**
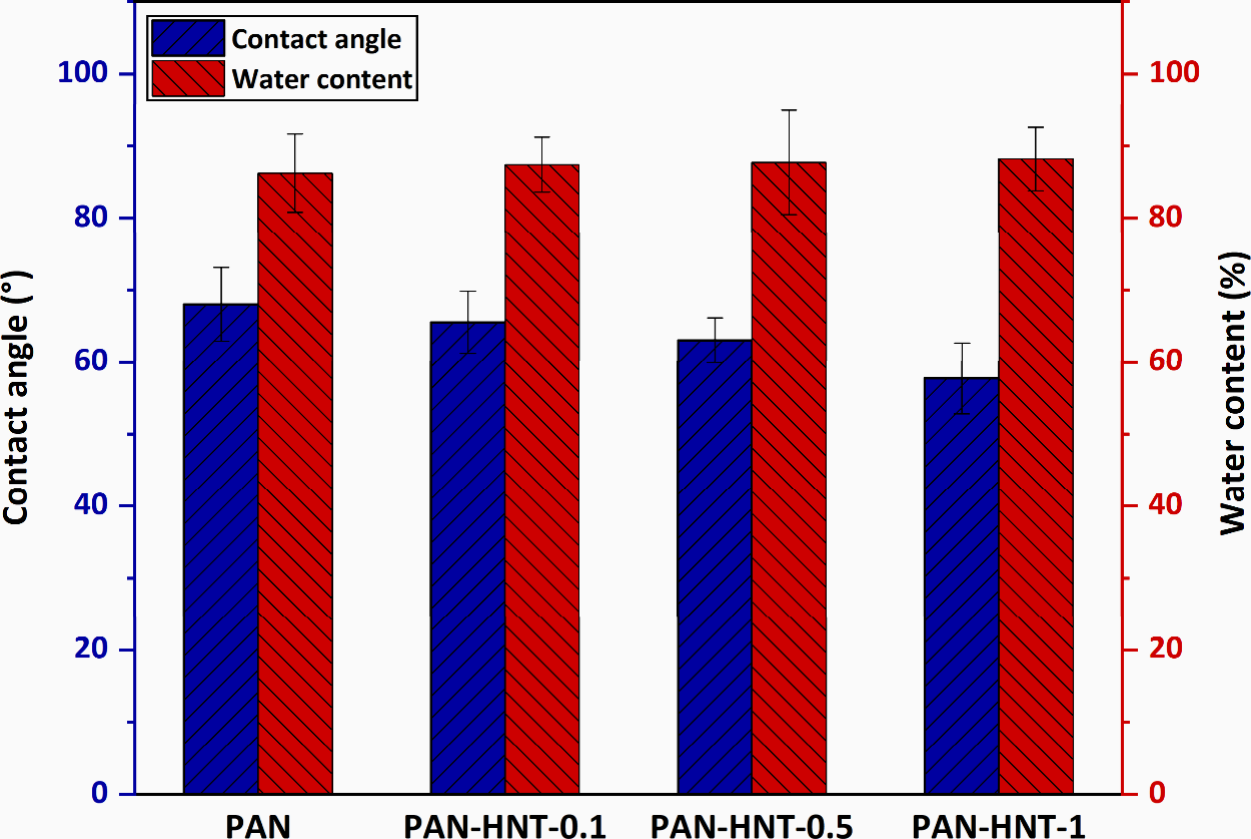
Contact angle and water content of neat PAN and HNT-doped PAN membranes.

Membrane surface hydrophilicity is characterized by contact angle measurement. In general, membranes with a surface contact angle less than 90° can be classified as hydrophilic while membranes greater than 90° can be classified as hydrophobic.^58^ The contact angle results of the produced membranes are listed in Figure 7. The contact angle of all produced membranes is less than 90°. That is, all membranes generally exhibited hydrophilic properties. Compared to the hydrophilicity of the membranes, the most hydrophobic membrane is neat PAN (68.0 ± 5.1°). As the HNT content increased in nanocomposite membranes, the contact angle decreased up to 57.7 ± 4.9° and the hydrophilicity of the membrane surface increased. The higher affinity of the hydrophilic −OH groups on the surface of the HNT to the water molecules dropped on the membrane surface caused the contact angle of the membranes to decrease.^30,36^ The membranes became more hydrophilic as the number of −OH groups increased and the amount of HNT included in the membrane increased. In addition, the lower surface hydrophilicity of the PAN membrane is consistent with the SEM images, showing that the surface of the PAN membrane is denser. In contact angle measurements, water that dropped on the membrane surface has more difficulty diffusing on a denser and less porous surface. This causes larger contact angle results to be obtained. Similarly, SEM surface views of HNT-doped nanocomposite PAN membranes are also consistent with their lower contact angles. The hydrophilic membrane surface reduces the interaction with hydrophobic contaminants and increases the membrane’s resistance to fouling.^79^ Therefore, based on the contact angle results, it can be said that the water filtration performance of the PAN membrane and its antifouling performance against hydrophobic contaminants in the water will increase with the addition of HNT.

Another parameter used to determine the hydrophilicity of the membranes is the water content of the membrane. The water content of all produced membranes is found to be over 85% (Figure 7). The water content of the PAN membrane (86.2% ± 5.4%) increased to 87.4% ± 3.8%, 87.7% ± 7.2%, and 88.2% ± 4.4% with the addition of 0.1, 0.5, and 1% wt HNT, respectively. The increase in the water content of the membrane by HNT can be explained by two factors: the hydrophilic character of HNT and the increase in membrane porosity. While the hydrophilic nature of the HNT increases the absorption of water, the high surface porosity of the membrane facilitates the penetration of water, increasing the water content of the membrane.

#### 3.1.6 Thermal Properties of Membranes

**Figure 8:**
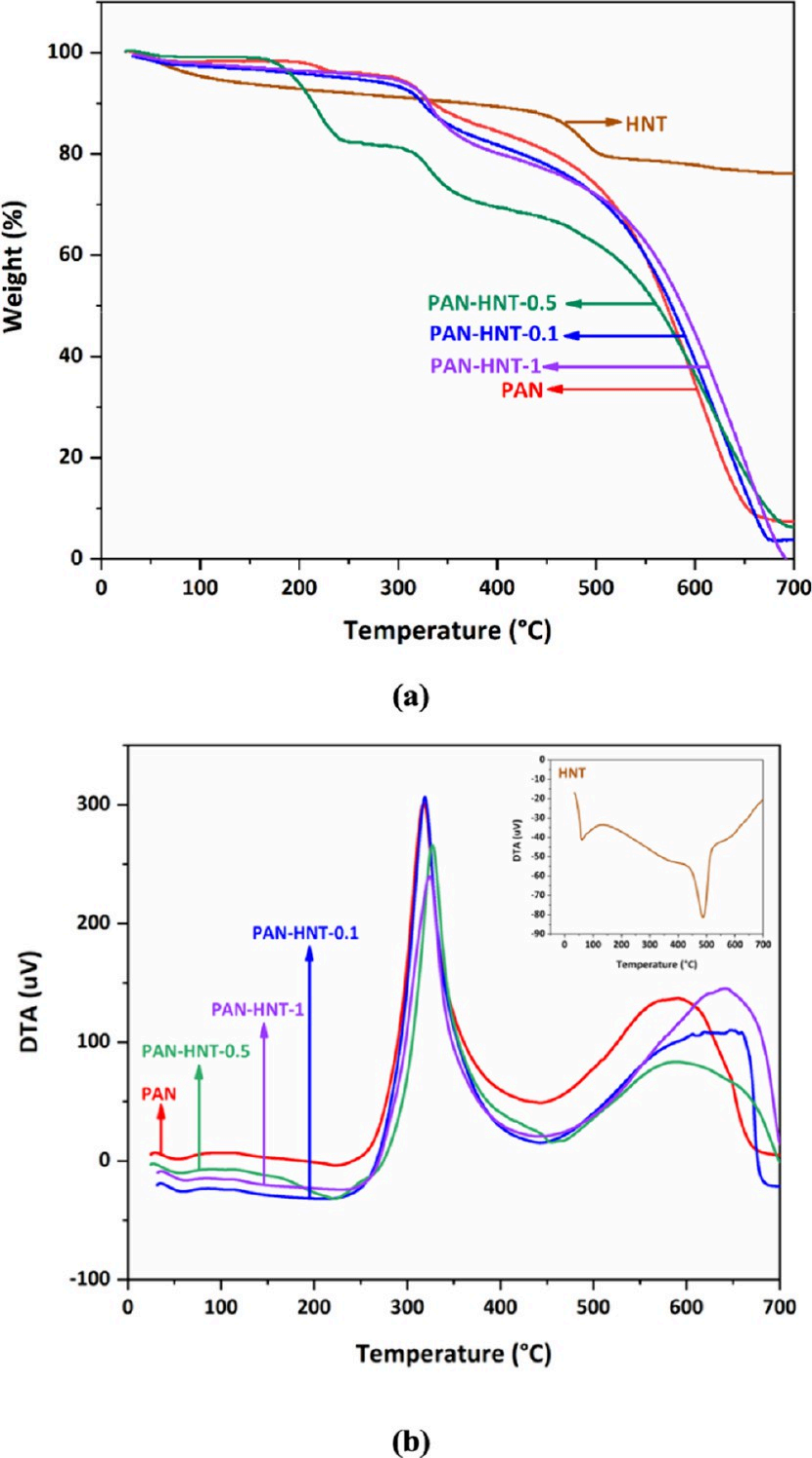
(a) TGA and (b) DTA curves of neat PAN, HNT, PAN-HNT-0.1, PAN-HNT-0.5, and PAN-HNT-1.

The thermal behavior of the membranes and HNT is evaluated, and the corresponding TGA/DTA curves are shown in Figure 8. The weight loss percentages of the membranes and HNT at different temperatures are listed in Table 3. A significant part of the weight loss for HNT occurs between 400 and 550 °C due to the dehydroxylation of structural water in AlOH groups.^80^ The weight losses of HNT are found to be 10.4, 19.3, and 21.9% at 400, 500, and 600 °C, respectively (Figure 8a). The maximum peak of HNT observed at 488 °C is assigned to dehydroxylation (Figure 8b). At 600 °C, PAN-HNT-0.1, PAN-HNT-0.5, and PAN-HNT-1 exhibited 60.1, 63.8, and 55.0% weight loss, respectively. The total weight of the PAN-HNT-1 membrane is completely lost at 700 °C (Figure 8a). If PAN is heated, chemical processes such as cyclization, degradation, and cross-linking can be observed. The cyclization of the nitrile group in PAN is an exothermic process.^81^ As seen in Figure 8b, the maximum peak is determined at 317 °C, which can be attributed to the cyclization of the nitrile group in the neat PAN membrane, and the broad second exothermic peak can be associated with the decomposition of PAN. Figure 8b also depicts that while the initiation temperature of the exothermic peak is around 240 °C and the maximum peak is determined at 318 °C for PAN-HNT-0.1, PAN-HNT-0.5 has the initiation temperature at 225 °C and the maximum peak at 328 °C. The maximum exothermic peak is observed at 324 °C for the PAN-HNT-1 membrane.

**Table 3:** Weight Loss Percentages of Prepared Membranes and the HNT for Various Temperatures

code	100 °C	200 °C	300 °C	400 °C	500 °C	600 °C	700 °C
HNT	4.3	6.9	8.5	10.4	19.3	21.9	23.6
Neat PAN	2.1	2.2	5.4	15.8	26.4	65.6	92.9
PAN-HNT-0.1	2.0	3.4	6.0	17.5	27.6	60.1	95.5
PAN-HNT-0.5	1.2	6.4	19.0	30.8	38.0	63.8	94.0
PAN-HNT-1	1.9	3.2	5.2	19.5	27.8	55.0	100.0

The weight loss of PAN-HNT-0.5 is the highest between 300 and 500 °C compared to other counterparts. The cyclization of nitrile in PAN-HNT-0.5 can appear to start at a lower temperature, and the maximum peak is observed at a slightly higher temperature. Considering FTIR spectra and SEM images, from which it can be confirmed that the halloysite is probably well dispersed in the PAN-HNT-0.5 membrane, thermal analysis results also showed that the PAN-HNT-0.5 membrane has a different thermal behavior between 300 and 500 °C. The higher addition of HNT further increased the thermal stability of PAN-HNT-1 at 600 °C. This can be assigned to the effect of a high surface area of the HNTs lumens.^67^ It is concluded that the contribution of HNT to the thermal stability of the membranes can depend on its critical ratio.

#### 3.1.7 Pure Water Flux of Membranes

**Figure 9:**
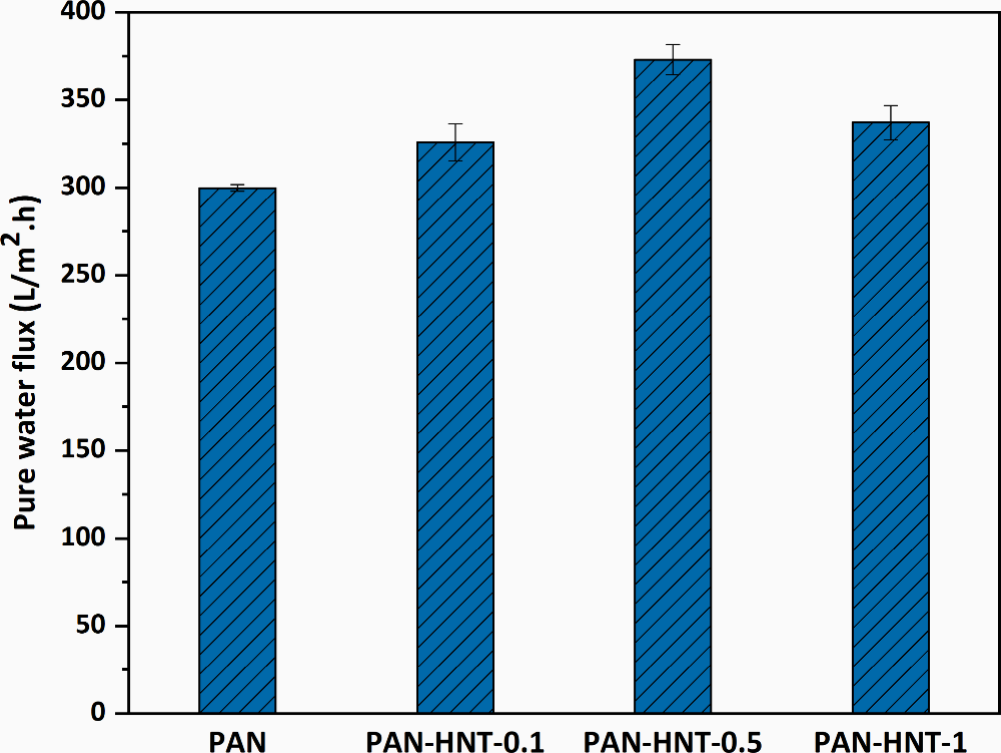
PWF of neat PAN and HNT-doped PAN membranes at 3 bar.

The PWF of the membranes under 3 bar of pressure is given in Figure 9. The lowest flux is obtained with a neat PAN membrane at 299.8 ± 2.1 L/m^2^ h. The PWF of the nanocomposite membranes is increased until the addition of 0.5% HNT by weight (373.1 ± 8.5 L/m^2^ h) and then decreased slightly. The PWF of the membranes with 0.1, 0.5, and 1% wt HNT is found to be 8.6, 24.4, and 12.4% higher, respectively, compared to the neat PAN membrane.

Membrane surface hydrophilicity and porosity are two important factors affecting the PWF of the membrane. The presence of hydrophilic −OH groups in the structure of the HNT contributed to the increase of the membrane surface hydrophilicity and porosity, increasing the affinity of the membrane against water and facilitating the passage of water through the membrane. On the other hand, the flux of the PAN-HNT-1 membrane (337 L/m^2^ h) is 9.6% less compared to the PAN-HNT-0.5 membrane. This result showed that the addition of a high amount of HNT into the PAN membrane casting solution increased the hydraulic resistance of the membrane due to the increase in viscosity, resulting in the formation of a denser and smaller pore-size membrane. In addition, a homogeneous dispersion of HNT without agglomeration in the high-viscosity casting solution is also very difficult.^38^ The failure to achieve the maximum benefit in the hydrophilic −OH groups of HNT, which could not be well dispersed in the membrane, also led to a decrease in the PWF performance. As a result, besides the hydrophilicity of the HNT itself, the physical changes caused by HNT in the membrane structure and the changes in the viscosity of the membrane casting solution by HNT are factors affecting the PWF performance. Therefore, HNT should be limited to the appropriate amount in the membrane.

### 3.2 Treatment Performance of PAN/HNT Membranes

**Figure 10:**
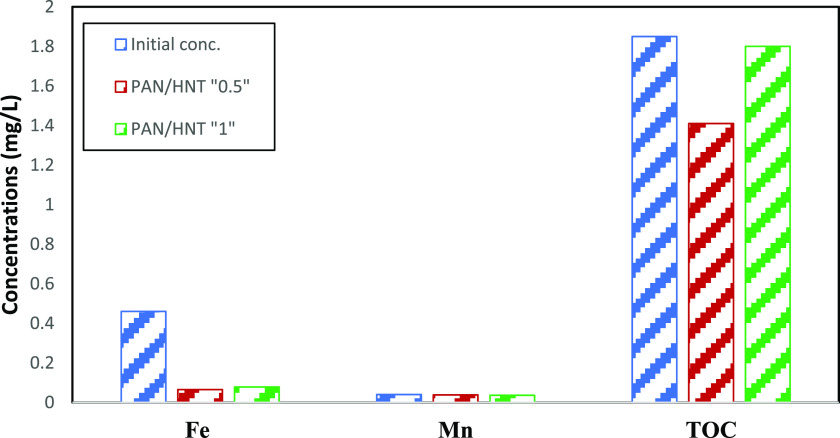
Filtration performances of membranes.

The change concentrations for Fe, Mn, and TOC after filtration of dam water through PAN-HNT-0.5 and PAN-HNT-1 membranes are given in Figure 10. Figure 10 shows that the initial Fe concentration of the dam water is 0.46 mg/L, the Mn concentration is 0.04 mg/L, and the TOC concentration is 1.85 mg/L. The dam water was passed through the PAN-HNT-0.5 and PAN-HNT-1 membranes to test membrane filtration. The Fe, Mn, and TOC concentrations of dam water that passed through the PAN-HNT-0.5 membrane decreased to 0.065, 0.038, and 1.41 mg/L, respectively. After passing through the PAN-HNT-1 membrane, the Fe, Mn, and TOC concentrations decreased to 0.078, 0.0363, and 1.8 mg/L, respectively. According to these results, the PAN-HNT-0.5 and PAN-HNT-1 membranes provided 85.8 and 83.0% efficiencies in Fe removal, respectively. In Mn removal, the PAN-HNT-0.5 and PAN-HNT-1 membranes provided 5.0 and 9.2% efficiencies, respectively. In TOC removal, the PAN-HNT-0.5 and PAN-HNT-1 membranes provided 23.7 and 2.7% efficiencies, respectively. According to these results, 0.5 wt % of HNT additive provided an advantage in Fe and TOC removal, while 1 wt % of HNT additive increased the efficiency in Mn removal. However, since the difference in Mn removal is not very significant, it is possible to say that the PAN-HNT-0.5 membrane is more suitable for treatment.

The high surface area of HNT makes it a good nanomaterial for the adsorption and further removal of contaminants in water. Due to the presence of HNT in the nanocomposite PAN-HNT-0.5 and PAN-HNT-1 membranes and their adsorption ability, the Fe^2+^, Mn^2+^, and TOC rejection performance of the nanocomposite membranes is higher than that of the PAN membrane. On the other hand, the properties of the membranes significantly affect the contaminant removal from the water. Because the surface porosity of the PAN-HNT-0.5 membrane is lower than that of the PAN-HNT-1 membrane (Figure 3), in general, contaminants pass through the PAN-HNT-0.5 membrane more difficultly. Contaminants are removed from the water at a higher rate as the passage through the membrane becomes more difficult (Figure 10). In addition, the electrostatic interaction between the membrane and contaminants also affects the removal efficiency of contaminants from water. The surface charge of HNT is negative at pH (7.39 ± 0.05) of the dam water used in this study.^82^ In this study, during the liquid–liquid exchange of the membranes produced by the phase inversion method, HNTs rise toward the membrane surface and accumulate on the surface. As a result, HNTs increase the surface negativity of the membrane.^36^ The electrostatic attraction between the negative membrane surface and positive Fe cations promotes the movement of Fe^2+^ toward the membrane surface. The high amount of HNT in the PAN-HNT-1 membrane may have increased the negativity of the membrane surface more and increased the passage of Fe^2+^ through the membrane by electrostatic attraction.

### 3.3 SEM Images of Fouled Membranes after Dam Water Filtration

**Figure 11:**
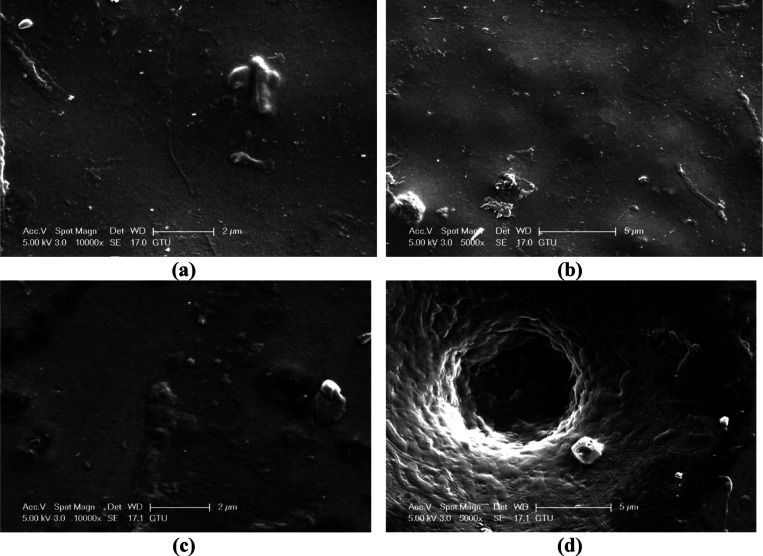
SEM surface images of (a, b) PAN-HNT-0.5 and (c)-(d) PAN-HNT-1.

The fouling on the membrane surface with filtration is closely related to the surface properties of the membrane and the contaminants in the feedwater. Membranes with high surface hydrophilicity and low surface roughness are more resistant to fouling. In this study, fouling on the surfaces of the membranes after filtration of dam water from the PAN-HNT-0.5 and PAN-HNT-1 membranes with high surface hydrophilicity, that is low contact angles, is investigated by SEM. Figure 11 shows the low- and high-magnification SEM images of the fouled PAN-HNT-0.5 and PAN-HNT-1 membranes. With the filtration of the dam water through the membranes, organic and inorganic contaminants in the dam water accumulated on both membrane surfaces, causing clogging of the open pores on the surfaces of the clean membranes (Figures 3 and 11). Membranes with high surface hydrophilicity exhibit a high affinity for water and, on the contrary, a low affinity for hydrophobic contaminants. Therefore, the high hydrophilicity of the membrane surface generally improves the antifouling property of the membrane surface. However, based on the data obtained from the contact angle measurements, although the surface hydrophilicity of PAN-HNT-1 is higher than that of PAN-HNT-0.5, fewer contaminants accumulated on the surface of PAN-HNT-0.5. The lower surface porosity of PAN-HNT-0.5 compared to PAN-HNT-1 makes it smoother, and the low number of ups and downs (pores) creates an unfavorable area for contaminants to accumulate on the membrane surface. Therefore, the combined effect of good surface hydrophilicity and low roughness made the PAN-HNT-0.5 membrane more resistant to fouling.

### 3.4 Mechanical Testing and Modeling

#### 3.4.1 Tensile Test

The tensile tests show the tensile strength, elongation at break, and elasticity modulus of each sample. In light of experimental studies, the HNT reinforcement effect and membrane behavior under various hygrothermal conditions can be reported, and results are given within the scope of this section.

**Figure 12:**
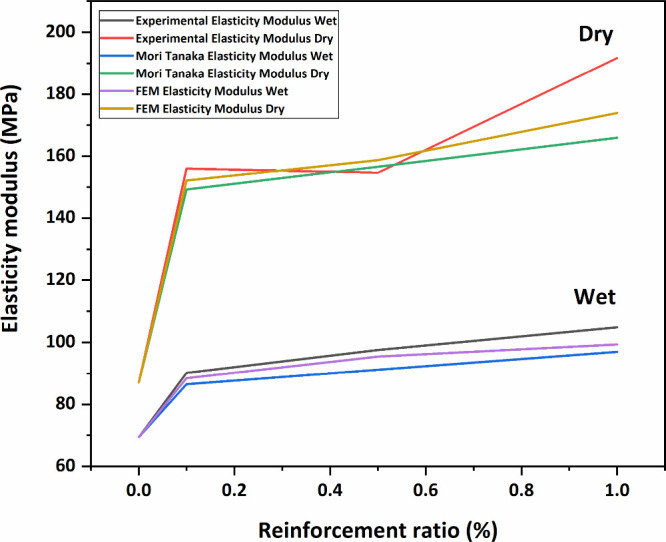
Calculated and measured elasticity modulus values.

Figure 12 presents the change in the modulus of elasticity of each membrane. From these results, it can obviously be said that with HNT reinforcement, the elasticity modulus of all membranes increases, and the membranes show a more rigid behavior. However, the increase in the modulus of elasticity with the linearly increasing HNT contribution is not linear. For example, a 29.8% increase in the modulus of elasticity of wet membranes is observed with 0.5% wt HNT additive and a rise of 40% is observed in the modulus of elasticity of wet membranes with 1% wt HNT additive. As a result, a nonlinear increase in the modulus of elasticity is observed with a linearly increasing HNT contribution.

**Figure 13:**
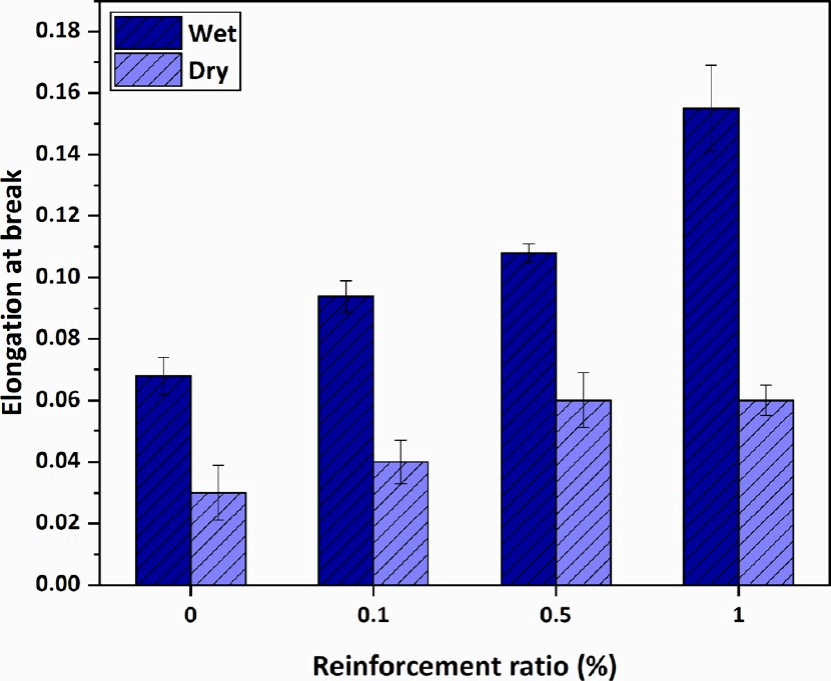
Elongation-at-break values of the wet and dry membranes.

Moreover, with increasing HNT reinforcement, an increase in the elongation at break values is observed. The first thing to note would be that the elongation at break values of wet membranes is higher than that of dry membranes. It can be pointed out that wet membranes have better ductility. Elongation-at-break values presented in Figure 13 also support this statement. Furthermore, when pure and 0.1% wt HNT-doped membranes are compared, the elongation-at-break values in the wet membrane case increase from 0.068 to 0.094. An increase in the number of dry membranes is also observed. When pure dry membranes and HNT-doped dry membranes are analyzed, an increment in elongation at break values can be detected, with values increasing from 0.03 to 0.06. When the increment ratios are evaluated in wet and dry membrane cases, 38.24, 58.82, and 127.94% increments in elongation-at-break values compared to the pure membrane are observed in HNT-doped wet membranes, respectively, and 33.33, 100, and 100% increments compared to the neat membrane are observed with HNT-doped dry membranes, respectively. This shows that HNT also has an increasing effect on membrane ductile behaviour, and the effect of the HNT reinforcement depends on the hygrothermal conditions.

Furthermore, upon comparing wet and dry membranes, higher elastic modulus values are determined in dry membranes, signifying a more rigid behavior.

**Figure 14:**
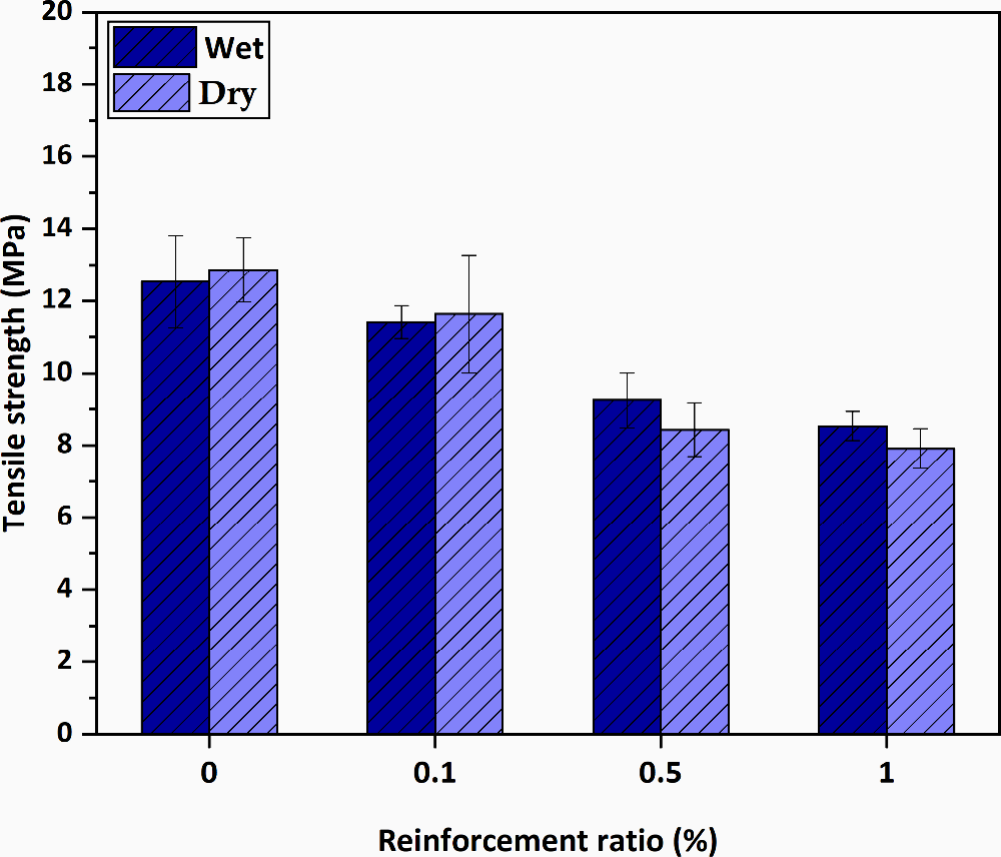
Tensile strength values of the tested samples.

The tensile strength properties acquired from the experimental procedure are displayed in Figure 14. From the results, it is seen that adding HNT decreases the membranes’ tensile strength properties. When the results are evaluated, it is seen that in the neat membrane case, the tensile strength values of the dry membranes are higher than those of the wet membranes, but the values are so close. Due to the HNT effect, the tensile strength values of 1 and 0.5% wt HNT-doped wet membranes are measured higher than those of dry membranes. Furthermore, when tensile strength changes are evaluated, 8.94, 26.18, and 31.92% decreases compared to the pure membrane are observed in HNT-doped wet membranes, respectively, and 9.49, 34.45, and 38.49% decreases compared to the neat membrane are observed with HNT-doped dry membranes, respectively. It can be said that the effect of HNT addition on tensile strength depends on the hygrothermal properties.

#### 3.4.2 Numerical Modeling

The homogenized mechanical properties of the composite membranes are determined using the Mori–Tanaka homogenization method and finite element analysis. The outcomes of each procedure are provided in distinct sections.

The HNT-doped PAN membranes of 0.1, 0.5, and 1% by weight are modeled with the Mori–Tanaka homogenization method. Their calculated elasticity modulus values are obtained as the output, and the results can be seen in Table 4.

**Table 4:** Experimental and Theoretical Determination of the Moduli of Elasticity

**condition**	**dry**			**wet**		
**reinforcement ratio (%)**	**experimental elasticity modulus [MPa]**	**Mori–Tanaka elasticity modulus [MPa]**	**FEM elasticity modulus [MPa]**	**experimental elasticity modulus [MPa]**	**Mori–Tanaka elasticity modulus [MPa]**	**FEM elasticity modulus [MPa]**
0.1	157.29	149.97	168.17	89.97	86.38	87.28
0.5	153.63	157.28	159.98	97.28	89.97	93.56
1.0	189.97	165.45	174.55	103.56	97.28	100.0

As a result of the analysis, the calculated elasticity modulus value of 0.1% by weight HNT-doped PAN membrane structures for wet conditions was 86.542 MPa and that for dry conditions was 149.27 MPa. In the 0.5% by weight HNT-reinforced PAN membrane case, the elasticity modulus is calculated for the wet condition, 91.156 MPa, and the dry state, 156.65 MPa. In the 1% by weight HNT-reinforced PAN membrane, the elasticity modulus is obtained for the wet condition of 96.97 MPa and the dry condition of 165.95 MPa.

In the analysis results, it is observed that the HNT reinforcement increases the elasticity modulus of the PAN membrane. The 0.1% wt HNT additive to the PAN membrane results in an increase of 1.35% in the wet condition and an increase of 1.23% in the dry case in the elasticity modulus of membranes. The 0.5% HNT additive leads to an increase of 6.75% in the wet condition and an increase of 6.26% in the dry case elasticity modulus values, and a 1% HNT additive increases the elasticity modulus values by 13.56% in the wet situation and 12.5634% in the dry condition.

From the results, it can be said that the 1% wt HNT-reinforced dry PAN membrane is the most rigid membrane in terms of mechanical loading.

The 0.1, 0.5, and 1% wt HNT-doped PAN wet and dry membranes are modeled with finite element analysis using FFT, and their predicted elasticity modulus values, stress, and strain distributions over RVE are obtained as program output; the results are given in this section.

The elasticity modulus values calculated with finite element analysis by using FFT are given in Table 4. From these results, with increasing HNT reinforcement, an increase in the elasticity modulus of composites is observed as an expected result. The increase rates in the elasticity modulus values are higher than the Mori–Tanaka results but close enough. Comparing these results with the Mori–Tanaka homogenization method, it can be said that with finite element analysis, more accurate results are obtained. However, when computational costs and results between these two methods are considered together, Mori–Tanaka homogenization results are also within an acceptable range. Besides, the Mori–Tanaka homogenization is computationally cheaper and can be computed faster.

**Figure 15:**
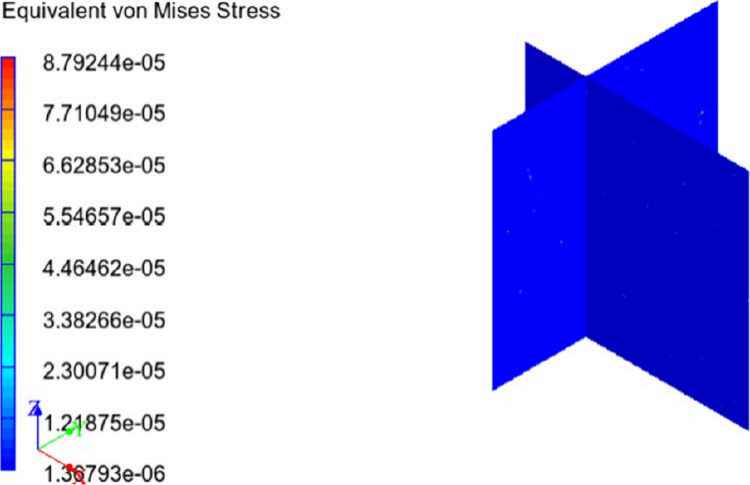
Equivalent von Mises stress distribution over RVE.

**Figure 16:**
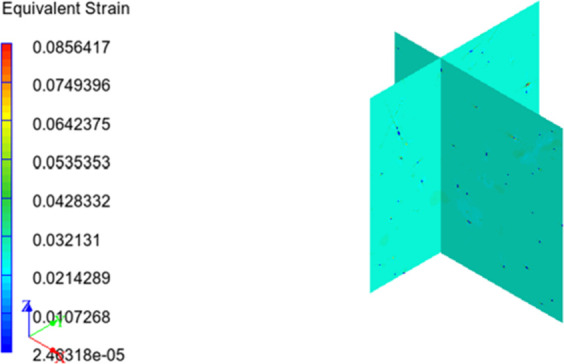
Equivalent strain distribution over RVE.

Besides, with finite element analysis, equivalent von Mises stress and strain distributions over the RVE can be obtained and distribution images of this study are presented in Figures 15 and 16,16. From the figures, it can be said that maximum equivalent stress and minimum equivalent strain occur on the reinforcing particulates. This may be due to the higher modulus of elasticity and rigidity of HNT content, and load is applied to the particles throughout the matrix phase.^58^

When the equivalent strain values are considered, it is seen that the displacement of the matrix phase is not much different compared with the composite structure. This can be attributed to the stress, modulus of elasticity, and strain relationship. It is known that the modulus of elasticity of the matrix material is very small compared with the reinforcement material. In addition, when the equivalent stress distribution is examined, it is seen that the reinforcement receives most of the load applied to the material. Considering the elasticity modulus, stress, and strain relationship, we can conclude that the strain distribution observed in the structure is also acceptable.

## 4 Conclusions

In this study, PAN and HNT-doped nanocomposite PAN UF membranes are fabricated by the phase inversion method and physical, chemical, thermal, and mechanical characterization of the membranes are performed. Experimental and numerical modeling procedures are followed to obtain the mechanical behavior of nanoparticle-doped membranes in hygrothermal conditions.

The results obtained in this study are listed below:

1. Even with a low amount of HNT (0.1% by weight) added to the PAN membrane, a change in the surface porosity and the number, size, and shape of finger-like pores in the inner structure of the membrane is observed, resulting in different membrane morphologies.
2. The hydrophilicity and water content of the PAN-HNT membranes are found to be higher than those of the neat PAN membrane due to HNT’s hydrophilic structure and increased membrane porosity. The membrane with the highest surface hydrophilicity (57.7 ± 4.9°) and water content (88.2% ± 4.4%) is determined as PAN-HNT-1.
3. PWF performance of the membrane is improved with the addition of HNT to the neat PAN membrane, which has the lowest PWF (299.8 ± 2.1 L/m^2^ h). The membrane with the highest flux is determined as the PAN-HNT-0.5 membrane (373.1 ± 8.5 L/m^2^ h), with an increase of 24.4% compared to the neat PAN membrane.
4. The HNT has an effect on the thermal stability of the membranes, which depends on the critical mass fraction ratio. However, the HNT particles are dispersed uniformly in the PAN-HNT-0.5 membrane and impact the thermal resistance in a positive way.
5. Fe, Mn, and TOC are removed from the dam water by the PAN-HNT-0.5 membrane at 85.8, 5.0, and 23.7%, respectively. With the PAN-HNT-0.5 membrane, Fe and TOC are removed from the dam water with an efficiency higher than that of PAN-HNT-1. The Mn removal rates of PAN-HNT-0.5 and PAN-HNT-1 are almost the same. In addition, it is observed that the PAN-HNT-0.5 membrane has higher antifouling ability compared to PAN-HNT-1 for contaminants in dam water. Therefore, we suggest that the PAN-HNT-0.5 membrane is the most suitable membrane for application in the treatment of dam water.

The 1% wt HNT-added dry PAN membrane is found to be the membrane with the highest elasticity modulus. From this result, it can be said that the 1% wt HNT-added dry PAN membrane is the most suitable membrane in terms of mechanical conditions. It can withstand the highest stress value. The results also indicate that the mechanical properties of the composite membranes are predictable and comparable. When homogenization results are examined, it can be said that the additive of HNT to polymer membranes increases the elasticity modulus of membranes. It is determined that the membrane that can withstand more load and is stiffer, and more suitable for mechanical performance is the 1% wt HNT added dry PAN membrane.

## Data Availability

Not applicable.
